# Switching to an alternative biological agent in juvenile idiopathic arthritis (I)

**DOI:** 10.1186/1546-0096-9-S1-P162

**Published:** 2011-09-14

**Authors:** A Remesal, S Murias, MI Gonzalez, I Tarjuelo, R Merino

## Background

Juvenile idiophatic arthritis (JIA) is a heterogeneous disease and it’s associated with an increased use of various biological agents in recent years.

## Aim

Describe changes in treatment with biological agents in JIA patients.

## Methods

This is a retrospective study of 109 JIA patients from a tertiary centre. Variables included were: age, disease duration, sex, antinuclear antibodies (ANA), HLA B27, Rheumatoid Factor (RF), uveitis and data of systemic AIJ. Current and previous biological agents, reasons for change and adverse events were also recorded.

## Results

The sample included 83 girls and 26 boys aged 10 ± 4.4 (2.4-19.6) with a disease duration of 5 ± 3.4 (0.1- 17) years. The initial biological agent was etanercept (ETA) in 87 cases, adalimumab (ADA) in 9, anakinra (AK) in 12 and infliximab (IFX) in 1. Twenty-eight patients switched to a second biological agent, 7 to a third and 2 to a fourth. Switching was due to inefficacy since adverse events were mild or moderate.

Current treatment with ETA was associated with ANA positives (p=0,032), however ADA did so with uveitis (p=0,000) and AK y TCZ with s-JIA (p=0,000). Table 1

**Table 1:** Patient characteristics and biological agent treatment

Current treatment	Sex M/F	ANA (+)	HLA B27 (+)	RF (+)	Uveitis	Data of s-JIA	Previous treatments
ETA n=58	11/47	19 (33%)	13 (22%)	2 (3%)	11 (19%)		ADA n=1

ADA n=16	2/14	6 (38%)	3 (19%)		14 (88%)	1 (7%)	ETA n=8 IFX n=1

AK n=9	5/4		1 (11%)			9 (100%)	ETA n=2

TCZ n=11	3/8		1 (9%)			6 (55%)	ETA n=7 IFX n=2 AK n=6 ADA n=3

IFX n=2	1/1		2 (100%)				ETA n=2

Untreated* n=13	4/9	2 (15%)	2 (15%)		1 (8%)	1** (8%)	ETA n=12 AK,TCZ n=1

* In 12 cases ETA was withdrawn due to inactive disease. **One child had episodes of macrophage activation syndrome, being with and without biological agent.

## Conclusion

1. Switching to a second biological agent was necessary in 26% of patients. Only 6% of patients switched to a third, and 2% to a fourth.

2. The results indicate that etanercept was chosen in patients with ANA positives, adalimumab in cases with uveitis and anakinra and tocilizumab in those with systemic symptoms.

